# KD_MultiSucc: incorporating multi-teacher knowledge distillation and word embeddings for cross-species prediction of protein succinylation sites

**DOI:** 10.1093/biomethods/bpaf041

**Published:** 2025-05-28

**Authors:** Thi-Xuan Tran, Thi-Tuyen Nguyen, Nguyen-Quoc-Khanh Le, Van-Nui Nguyen

**Affiliations:** Faculty of Foundation Studies, Thai Nguyen University of Economics and Business Administration, Thai Nguyen City, 2500000, Vietnam; Faculty of Information Technology, Thai Nguyen University of Information and Communication Technology, Thai Nguyen City, 2500000, Vietnam; Professional Master Program in Artificial Intelligence in Medicine, College of Medicine, Taipei Medical University, Taipei, 110, Taiwan; AIBioMed Research Group, Taipei Medical University, Taipei, 110, Taiwan; Faculty of Information Technology, Thai Nguyen University of Information and Communication Technology, Thai Nguyen City, 2500000, Vietnam

**Keywords:** succinylation, bi-direction long short-term memory (Bi-LSTM), convolutional neural network (CNN), natural language processing (NLP), word embedding, knowledge distillation

## Abstract

Protein succinylation is a vital post-translational modification (PTM) that involves the covalent attachment of a succinyl group (-CO-CH2-CH2-CO-) to the lysine residue of a protein molecule. The mechanism underlying the succinylation process plays a critical role in regulating protein structure, stability, and function, contributing to various biological processes, including metabolism, gene expression, and signal transduction. Succinylation has also been associated with numerous diseases, such as cancer, neurodegenerative disorders, and metabolic syndromes. Due to its important roles, the accurate prediction of succinylation sites is essential for a comprehensive understanding of the mechanisms underlying succinylation. Although research on the identification of protein succinylation sites has been increasing, experimental methods remain time-consuming and costly, underscoring the need for efficient computational approaches. In this study, we present KD_MultiSucc, a model for cross-species prediction of succinylation sites using Multi-Teacher Knowledge Distillation and Word Embedding. The proposed method leverages the strengths of both Knowledge Distillation and Word Embedding techniques to reduce computational complexity while maintaining high accuracy in predicting protein succinylation sites across species. Experimental results demonstrate that the proposed predictor outperforms existing predictors, providing a valuable contribution to PTM research and biomedical applications. To assist readers and researchers, the codes and resources related to this work have been made freely accessible on GitHub at https://github.com/nuinvtnu/KD_MultiSucc/.

## Introduction

Post-translational modification (PTM) is a chemical regulatory mechanism that takes place after protein translation. This regulation is crucial for various cellular processes, including signaling networks, protein degradation, gene transcription regulation, protein–protein interactions, and metabolic pathways. Most PTMs are driven by the enzymatic attachment or removal of chemical groups [1]. Protein succinylation is a vital PTM that involves the covalent attachment of a succinyl group to lysine residues in proteins. The mechanism underlying succinylation process plays a critical role in regulating protein structure, stability, and function [2–7]. It is involved in various biological processes, including metabolism, gene expression, and signal transduction, and has been associated with numerous diseases such as cancer, neurodegenerative diseases, and metabolic disorders, highlighting its potential as a biomarker and therapeutic target. Due to its important roles, accurate prediction of succinylation sites is essential for understanding the molecular mechanisms underlying these processes and for facilitating drug discovery and therapeutic development. Recently, advances in high-throughput proteomics and computational modelling have facilitated the identification and prediction of succinylation sites, contributing to a deeper understanding of its biological significance. Despite advances in experimental methods, they remain time-consuming and costly, underscoring the need for efficient computational approaches.

Due to the important roles of succinylation mechanisms, various computational prediction tools have been developed to identify succinylation sites, with a particular focus on species-specific and generic succinylation data [8–18]. SuccinSite 2.0 [12] is an updated tool that incorporates a larger dataset and enhanced algorithms to improve prediction accuracy for multiple species, including *Homo sapiens*, *Escherichia coli*, and *Saccharomyces cerevisiae*. It provides user-friendly features for species-specific predictions, making it valuable for researchers working on specific organisms. The method combines two key sequence features: profile-based composition of k-spaced amino acid pairs (pCKSAAP) and binary amino acid codes (BE), which are processed by a Random Forest (RF) classifier to integrate the classification results from different models. Similarly, GPSuc [10] is notable for its broad application across multiple species, leveraging a combination of traditional features and logistic regression to achieve robust predictive performance. Both tools highlight the growing need for species-specific datasets and the importance of incorporating diverse features into predictive models.

In recent years, deep learning-based methods have emerged for predicting succinylation sites, particularly focusing on generic data. For example, in [11], Thapa *et al*. developed DeepSuccinylSite, a CNN-based model using a hybrid of one-hot encoding and an embedding layer for sequence representation, which bypasses the need for manual feature extraction. Similarly, in [16], Zhang and Wang proposed DeepSuc, a hybrid CNN-LSTM model, but it relies on pre-extracted features such as CKSAAP, ACF, and BLOSUM62 instead of learning directly from raw data. Meanwhile, Ning *et al*. [9] introduced HybridSucc, which combines DNN and PLR using seven types of sequence-derived features, although this method also requires feature engineering. In [8], Huang *et al*. utilized Word2Vec embedding with a hybrid LSTM-CNN model (LSTMCNNsucc), but the Word2Vec features had to be pre-extracted prior to training, adding computational overhead. Another notable approach is by Pokharel *et al*. [19], who integrated supervised Word Embedding and embedding from the ProtT5-XL-UniRef50 protein language model into an artificial neural network. While this method effectively captures sequence-specific patterns using embedding from a pre-trained language model, it has limitations. For instance, the ProtT5 embedding are high-dimensional (1024 per amino acid) and computationally expensive to generate, making it less efficient for large-scale applications.

Despite the advances in these deep learning models, many of them still rely heavily on manual feature extraction or pre-trained embedding, which can be computationally intensive and may limit scalability. Inspired by the Knowledge Distillation mechanism [20–28], we propose a model named **KD_MultiSucc** to address these challenges. This approach incorporates Multi-Teacher Knowledge Distillation (KD) and Word Embedding to predict protein succinylation sites across multiple species, reducing computational complexity while maintaining high accuracy. The Multi-Teacher KD architecture can leverage the complementary strengths of CNN and Bi-LSTM models as Teacher networks. This allows the Student model to distil and integrate high-quality knowledge from both Teachers while learning directly from raw sequence data. Besides, by employing Word Embedding to encode amino acid sequences, our method reduces the dependency on pre-extracted features and achieves high predictive performance with lower computational overhead.

Experimental results demonstrated that the proposed model, **KD_MultiSucc,** has been rigorously evaluated on benchmark datasets, showcasing superior performance compared to existing methods. This study not only advances succinylation site prediction but also highlights the effectiveness of combining KD and NLP-based embedding in bioinformatics, offering a scalable and efficient solution for PTM analysis.

## Materials and methods

### Data preparation and pre-processing

In this study, we aim to develop an architecture for predicting succinylation sites in both generic and species-specific contexts. The dataset, sourced from SuccinSite2.0 [12] and GPSuc [10], mirrors their training and test sets to ensure consistency in model development and comparison. It includes data from nine species: *Homo sapiens (H. sapiens), Mus musculus (M. musculus), Escherichia coli (E. coli), Mycobacterium tuberculosis (M. tuberculosis), Saccharomyces cerevisiae (S. cerevisiae), Toxoplasma gondii (T. gondii), Solanum lycopersicum (S. lycopersicum)*, and others. The detailed information of the collected datasets is summarized in Table 1.

**Table 1.** Summary of data collection in this study.

Species	Dataset	SuccinSite2.0		GPSuc	
		Number of proteins	Positive sites	Number of proteins	Positive sites
Generic	Training	2,198	4,750	2198	4,750
	Testing	124	254	124	254
*H. sapiens*	Training	500	1,351	500	1,351
	Testing	50	54	50	54
*H. capsulatum*	Training	–	–	150	332
	Testing	–	–	33	50
*M. musculus*	Training	240	414	240	414
	Testing	24	24	24	24
*E. coli*	Training	786	1,942	786	1,942
	Testing	79	289	79	289
*M. tuberculosis*	Training	369	699	369	699
	Testing	36	61	36	61
*S. cerevisiae*	Training	364	961	364	961
	Testing	36	90	36	90
*T. gondii*	Training	98	282	98	282
	Testing	10	26	10	26
*S. lycopersicum*	Training	150	242	150	242
	Testing	16	33	16	33
*T. aestivum*	Training	–	–	53	113
	Testing	–	–	20	32

Since this study focuses on the sequence-based characterization of lysine (K) succinylation sites and their substrate specificities, it is necessary to extract sequence fragments from the full FASTA sequence of proteins. Consistent with previous research, positive samples were constructed by extracting sequence fragments using a window size of 2n+1, centering the lysine (K) residue that has been experimentally verified as a succinylation site, with n upstream and n downstream residues. For fragments with fewer than n residues on either side, the pseudo-amino acids (‘X’) were added to standardize the length to 2n+1. Based on GPSuc [10] and our experimental analysis, the optimal window size was found to be 33; therefore, we adopted the same approach in this study.

For negative samples (non-succinylation sites), lysine residues not experimentally verified as succinylation sites were treated as non-succinylation sites, and the same window size of 2n+1 was applied to extract negative fragments. To mitigate overfitting and enhance the model’s generalization, sequence fragments were filtered using CD-HIT with a 30 per cent identity cut-off. The final datasets used in this study are summarized as in Table 2.

**Table 2.** Detailed information of the datasets used in this study.

Species	Dataset	Succinylated proteins	Succinylation sites	Non-Succinylation sites
Generic	Training	2,198	4,750	9,500
	Testing	124	254	2,977
*H. sapiens*	Training	500	1,351	2,702
	Testing	50	54	2,004
*H. capsulatum*	Training	150	332	664
	Testing	33	50	591
*M. musculus*	Training	240	414	828
	Testing	24	24	679
*E. coli*	Training	786	1942	3,884
	Testing	79	289	1,381
*M. tuberculosis*	Training	369	699	1,398
	Testing	36	61	242
*S. cerevisiae*	Training	364	961	1,922
	Testing	36	90	1,423
*T. gondii*	Training	98	282	564
	Testing	10	26	261
*S. lycopersicum*	Training	150	242	484
	Testing	16	33	274
T*. aestivum*	Training	53	113	226
	Testing	20	32	309

As displayed in Table 2, the Generic dataset consists of 2,198 training proteins and 124 test proteins, with 4,750 succinylation sites and 9,500 non-succinylation sites in the training set, and 254 succinylation sites, and 2,977 non-succinylation sites in the test set. The species-specific datasets are varied in size: for example, the *S. cerevisiae* dataset contains 961 succinylation sites and 1,922 non-succinylation sites for training, with a test set of 90 succinylation sites and 1,423 non-succinylation sites. Smaller datasets, such as for *T. gondii and S. lycopersicum*, illustrate the challenges posed by limited data. The detailed information of the dataset used in this study is summarized in Table 1 below.

### Feature engineering

After preparing the final datasets, the feature engineering process was performed to standardize and extract features from protein sequences for input into the deep learning model. This process was summarized in three steps as follows:

#### Step 1. Sequence fragments representation

Each sequence fragment was represented as a string of characters corresponding to amino acids (e.g., MDKETVELEAELNQLKEENAQLKHALAPSAVEA). The sequence fragment was then divided into unigram units, treating each amino acid as an independent element (e.g., K, Q, A, Y, …). This step simplifies the sequence fragment structure by breaking it down into its fundamental components, facilitating easier processing by the model.

#### Step 2. Tokenization using 1-gram

After step 1, the amino acids were converted into token through a tokenization process, utilizing a vocabulary consisting of 20 types of amino acids (‘A’, ‘C’, ‘D’, ‘E’, ‘F’, ‘G’, ‘H’, ‘I’, ‘K’, ‘L’, ‘M’, ‘N’, ‘P’, ‘Q’, ‘R’, ‘S’, ‘T’, ‘V’, ‘W’, and ‘Y’) and a special character like ‘X’ representing unidentified amino acids. This step transformed the sequence fragment into the token in the form of a vector $\left(x_{1},x_{2},\;\ldots ,\;x_{L}\right)$*,* where L is the length of the sequence fragment.

#### Step 3. Embedding

After tokenization process, the embedding layer was applied to transform the token $\left(x_{1},x_{2},\;\ldots ,\;x_{L}\right)$ into numerical vector $\left(v_{1},v_{2},\;\ldots ,\;v_{L}\right)$ with semantic and spatial significance, where $v_{i}\in R^{d}$ and *d* is the vector dimension. Since the predictive models are designed based on the integration of three baseline architectures (CNN1D, Bi-LSTM, and CNN1D+Bi-LSTM), we decided to investigate the performance of these baseline models on the Generic dataset with different common values of embedding dimension (50, 100, 150, 200, 250, 300, …) to determine the optimal embedding dimension for our model. In addition, we also considered the trade-offs between model complexity and performance. Finally, the optimal value of *d* was determined to be 300 for this study.

**Figure 1.**
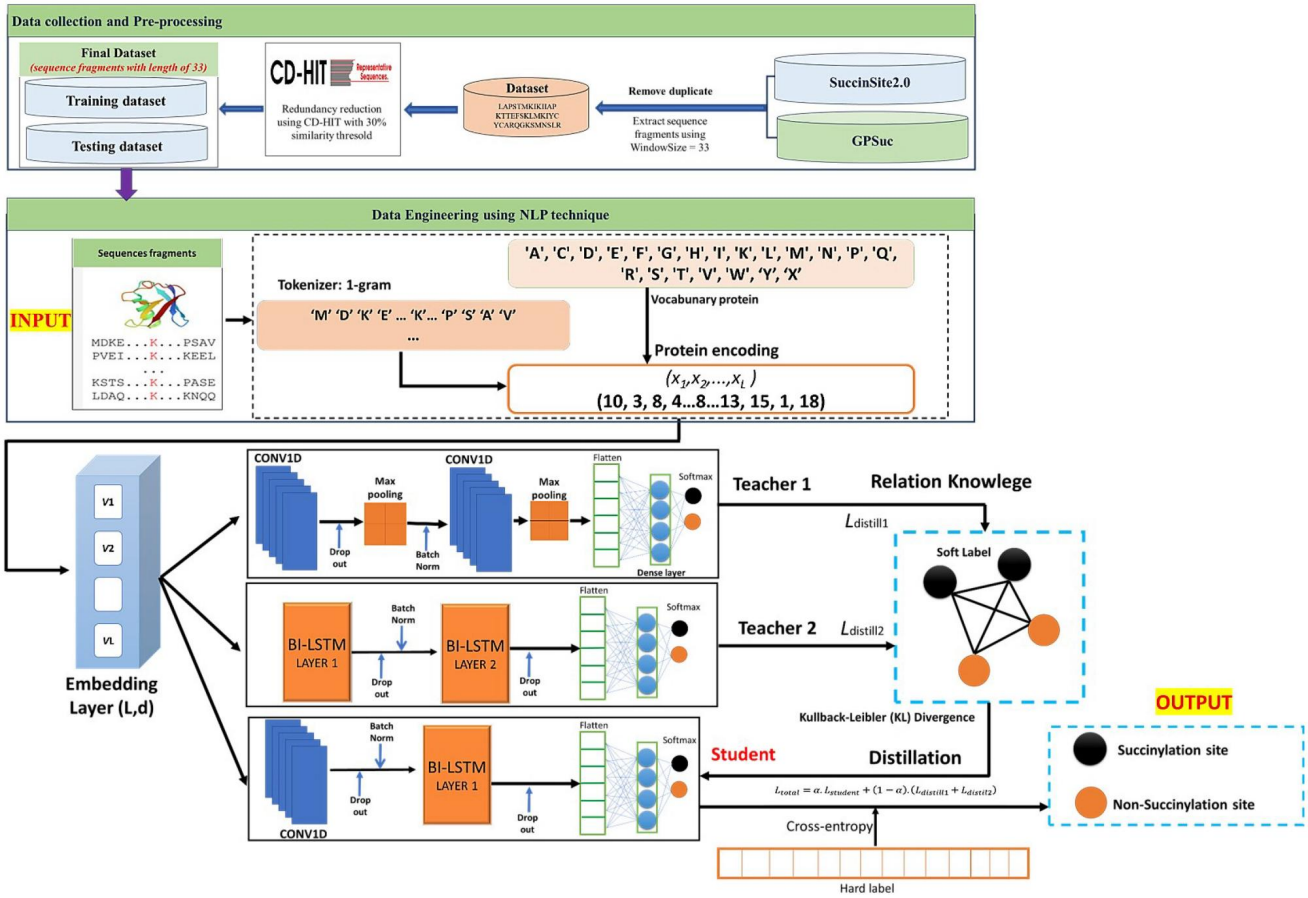
System architecture of **KD_MultiSucc**: A hybrid Student model combining CNN1D and Bi-LSTM, trained under multi-teacher knowledge distillation with CNN1D and Bi-LSTM Teachers

The final result of the process was a matrix of embedded vector with size of *(L, d)*, which was then fed into the deep learning model. Hidden layers in the model further extracted complex features to support the prediction of protein characteristics or succinylation sites. This process ensures that the data is represented in the most suitable form for optimal model performance. The details information of the process is displayed in Fig. 1.

### Evaluation metrics

To evaluate the performance of the predictive models, we employed a 10-fold cross-validation approach to assess the classification power of the predictive models. The following metrics were used to evaluate model performance: Sensitivity (SEN), Specificity (SPE), Accuracy (ACC), and Matthew’s correlation coefficient (MCC).


(1) $$\mathrm{Sensitivity}\;(\mathrm{SEN})=\frac{TP}{TP+FN}$$

(2) $$\mathrm{Specificity}\;(\mathrm{SPE})=\frac{TN}{TN+FP}$$

(3) $$\mathrm{Accuracy}\;(\mathrm{ACC})=\frac{TP+TN}{TP+\mathrm{FP}+\mathrm{TN}+FN}$$

(4) $$(\mathrm{MCC})=\frac{\left(TP\times TN)-(FN\times FP\right)}{\sqrt{\left(TP+FN)\times (TN+FP)(TP+FP)(TN+FN\right)}}$$

Wherein TP, TN, FP, and FN represent the numbers of true positives, true negatives, false positives, and false negatives, respectively. Sensitivity measures the rate of correct predictions for positive data, while specificity does the same for negative data. Accuracy represents the overall proportion of correctly predicted positive and negative instances. However, in binary classification, accuracy may be misleading when class sizes are highly imbalanced. Therefore, MCC is often preferred as a more balanced metric, even when class sizes differ significantly. MCC ranges from −1 to +1, whereas sensitivity (SEN), specificity (SPE), and accuracy (ACC) range from 0 to 1. An MCC of +1 indicates perfect predictions, while values of 0 and −1 correspond to random and completely incorrect predictions, respectively. In general, a higher positive MCC value reflects better classification performance for both positive and negative data. In addition, to visualize the output probability distributions of both Teacher and Student models, the t-distributed stochastic neighbor embedding (t-SNE) [29] has been conducted (e.g., both datasets: Generic and *H. sapiens*) to illustrate the knowledge transfer between the models, as well as to evaluate the efficiency of knowledge transfer from Teacher to Student models.

In order to evaluate the performance of the predictive models, the k-fold cross-validation and independent test approaches were used. Using the 10-fold cross-validation approach, the predictive model with the highest accuracy and MCC values was selected as the final model for predicting protein succinylation sites in this study. Additionally, an independent approach was conducted using a testing set to evaluate the model’s performance in real-world scenarios.

### Model learning and evaluation

In our study, we employed a dual-teacher KD framework using CNN1D and Bi-LSTM as based models. This choice was motivated by both biological characteristics of protein sequences and the proven effectiveness of these architectures in PTM site prediction. CNN1Dwere effectively capable of capturing spatial and local pattern features, making them suitable for tasks such as PTM prediction [30–32], protein function prediction [33, 34], and DNA analysis [35]. Meanwhile, Bi-LSTM excels at learning sequential dependencies and contextual information in time-series or sequential data [8, 36]. To leverage the strengths of both architectures, we conducted experiments to evaluate the performance of three baseline architectures: CNN1D, Bi-LSTM, and CNN1D_Bi-LSTM, before proposing our Multi-Teacher KD approach.

Based on the experimental results presented in Table 3, the selection of the Multi-Teacher KD architecture was based on the performance of the baseline networks to optimize prediction efficiency. The CNN1D network demonstrates superior non-sequential feature extraction, with high specificity in most datasets. For example, on the *E. coli* dataset, the accuracy (ACC) is 0.78, and the MCC is 0.50. However, the CNN1D tends to exhibit lower sensitivity compared to the Bi-LSTM. In contrast, the Bi-LSTM shows better sequential feature extraction, achieving high sensitivity on datasets such as *H. sapiens*, *S. cerevisiae*, and *T. aestivum*. However, in some cases, such as with *H. sapiens*, the accuracy and MCC are lower than those of the CNN1D. The hybrid network of CNN+Bi-LSTM demonstrates a good balance between specificity and sensitivity, with superior MCC results across multiple datasets, notably *H.capsulatum* (MCC = 0.36). Based on these analyses, CNN1D was selected as Teacher 1 due to its ability to extract non-sequential features, while Bi-LSTM was selected as Teacher 2 for its superior capability in handling sequential information. The CNN1D+Bi-LSTM architecture was selected as Student because of its ability to combine the advantages of both Teachers, helping to optimize the learning efficiency from diverse information sources.

**Table 3.** Ten-fold cross-validation performance of the three baseline architectures.

Dataset	CNN1D				Bi-LSTM				CNN1D+Bi-LSTM			
	SPE	SEN	ACC	MCC	SPE	SEN	ACC	MCC	SPE	SEN	ACC	MCC
Generic	0.84	0.61	0.77	0.46	0.80	0.62	0.74	0.43	0.80	0.69	0.77	0.46
*H. sapiens*	0.84	0.61	0.78	0.49	0.82	0.64	0.75	0.43	0.84	0.63	0.78	0.49
*H. capsulatum*	0.83	0.42	0.69	0.26	0.82	0.44	0.70	0.29	0.87	0.49	0.73	0.36
*M. musculusis*	0.84	0.50	0.75	0.43	0.82	0.51	0.72	0.33	0.86	0.53	0.75	0.42
*E. coli*	0.84	0.65	0.78	0.50	0.83	0.66	0.76	0.46	0.83	0.63	0.76	0.46
*M. tuberculosis*	0.79	0.37	0.65	0.18	0.78	0.38	0.64	0.16	0.79	0.36	0.65	0.17
*S. cerevisiae*	0.83	0.58	0.77	0.47	0.84	0.66	0.75	0.41	0.86	0.58	0.77	0.46
*T. gondii*	0.82	0.41	0.67	0.24	0.81	0.42	0.68	0.24	0.83	0.39	0.68	0.24
*S. lycopersicum*	0.82	0.43	0.69	0.27	0.84	0.47	0.71	0.32	0.81	0.52	0.71	0.34
*T. eastivum*	0.83	0.36	0.61	0.10	0.82	0.42	0.70	0.29	0.84	0.46	0.71	0.32

In this study, we conducted experiments using four Multi-Teacher KD architectures: KD1 *(Teacher 1: CNN1D, Teacher 2: Bi-LSTM, Student: CNN1D)*, KD2 *(Teacher 1: CNN1D, Teacher 2: Bi-LSTM, Student: Bi-LSTM)*, KD3 *(Teacher 1: CNN1D, Teacher 2: Bi-LSTM, Student: CNN1D+Bi-LSTM)*, and KD4 *(Teacher 1: CNN1D, Teacher 2: Bi-LSTM, Student: Bi-LSTM+CNN1D)*. The goal of this selection was to comprehensively evaluate the knowledge synthesis ability and learning efficiency of different Student models:

KD1 and KD2 were designed as controlled experiments, in which the Student model shares architectural similarity with only one of the two teachers. This enables us to isolate the individual influence of each teacher on the student’s learning process and measure how effectively a CNN1D or Bi-LSTM-based student can absorb knowledge distilled from both teachers. These configurations serve as baselines to assess the relative benefits of architecture alignment in the knowledge transfer process.

In contrast, KD3 and KD4 explore hybrid student architectures trained under the same dual-teacher setting. While both use combined CNN1D and Bi-LSTM components, they differ in the order of integration: KD3 follows a sequential pipeline of CNN1D followed by Bi-LSTM (CNN1D→Bi-LSTM), focusing first on extracting local sequence motifs before modeling long-range dependencies; KD4 adopts the reverse order (Bi-LSTM→CNN1D), capturing global contextual patterns first, then refining local features. This design allows us to assess not only the benefit of hybrid learning from heterogeneous teachers but also how the sequencing of components in the student architecture affects the integration and utilization of multi-source knowledge.

The configurations of the four Multi-Teacher KD architectures are summarized in Table 4.

**Table 4.** The configurations of the four multi-teacher knowledge distillation architectures.

Model	Category	Parameter	Value
KD1	Teacher 1	CNN1D	2 layers, filters = 32, kernel_size = 3, activation = 'relu'
	Teacher 2	Bi-LSTM	2 layers, units = 32, return**_**sequences = True
	Student	CNN1D	1 layer of CNN1D (filters = 32, kernel_size = 3, activation = 'relu'); 1 layer of Bi-LSTM (units = 32, return_sequences = True)
	**Training parameters**: Alpha (α): 0.5, Temperature (τ): 10, Learning Rate: 0.0001, Dropout: 0.4, Optimizer: Adam, Epochs: 50, Batch_size: 16, Embedding_layer (Embeding_dim: 300, vocab_size: 21, **input_length = max_length, Trainable = True**)		
KD2	Teacher 1	CNN1D	2 layers, filters = 32, kernel_size = 3, activation = 'relu'
	Teacher 2	Bi-LSTM	2 layers, units = 32, return**_**sequences =True
	Student	Bi-LSTM	1 layer of CNN1D (filters = 32, kernel_size = 3, activation = 'relu'); 1 layer of Bi-LSTM (units = 32, return_sequences = True)
	**Training parameters**: Alpha (α): 0.5, Temperature (τ): 10, Learning Rate: 0.0001, Dropout: 0.4, Optimizer: Adam, Epochs: 50, Batch_size: 16, Embedding_layer (Embeding_dim: 300, vocab_size: 21, **input_length = max_length, Trainable** **=** **True**)		
KD3	Teacher 1	CNN1D	2 layers, filters = 32, kernel_size = 3, activation = 'relu'
	Teacher 2	Bi-LSTM	2 layers, units = 32, return_sequences = True
	Student	CNN1D+ Bi-LSTM	1 layer of CNN1D (filters = 32, kernel_size = 3, activation = 'relu'); 1 layer of Bi-LSTM(units = 32, return_sequences = True)
	**Training parameters**: Alpha (α): 0.5, Temperature (τ): 10, Learning Rate: 0.0001, Dropout: 0.4, Optimizer: Adam, Epochs: 50, Batch_size: 16, Embedding_layer (**Embedding_dim: 300, vocab_size: 21, input_length = max_length, trainable = True**)		
KD4	Teacher 1	CNN1D	2 layers, filters = 32, kernel_size = 3, activation = 'relu'
	Teacher 2	Bi-LSTM	2 layers, units = 32, return_sequences = True
	Student	Bi-LSTM+ CNN1D	1 layer of Bi-LSTM(units = 32, return_sequences = True); 1 layer of CNN1D (filters = 32, kernel_size = 3, activation = 'relu')
	**Training parameters**: Alpha (α): 0.5, Temperature (τ): 10, Learning Rate: 0.0001, Dropout: 0.4, Optimizer: Adam, Epochs: 50, Batch_size: 16, Embedding_layer (**Embedding_dim: 300, vocab_size: 21, input_length = max_length, trainable** **=** **True**)		

Although our proposed KD3 configuration involves two teacher networks—Teacher 1 (CNN1D) and Teacher 2 (Bi-LSTM)—and the student model integrates both architectural styles, the computational complexity remains well-controlled.

As shown in Table 5, Teacher 1 contains 53,130 parameters and Teacher 2 has 252,298 parameters, totaling 305,428 parameters and approximately 69,308 KB of memory usage. In contrast, the student model comprises only 117,802 parameters with a memory footprint of 46,016 KB. These results confirm that the student model is substantially smaller and more efficient than the combined teachers. Therefore, although we utilize two teachers to transfer complementary knowledge—motif learning via CNN1D and sequence feature extraction via Bi-LSTM—the resulting student model maintains a good balance between representational capacity and resource efficiency.

**Table 5.** Comparison of model complexity in terms of total parameters and memory consumption between Teacher and Student models.

Model	Params	SUM	Memory (KB)	SUM (KB)
Teacher 1	53,130	305,428	20,754	69,308
Teacher 2	252,298		48,554	
Student	117,802	117,802	46,016	46,016

The architecture of the proposed model was constructed based on an idea of the combination between KD model and Word Embedding. The detail of the architecture’s formation was summarized as follows:

- ***Teacher models:***
  - The architecture of the predictive models includes two Teacher models: one Teacher is a multi-layer 1D Convolutional Neural Network (CNN1D) designed to extracts spatial features from the protein sequences, while other Teacher is a multi-layer Bi-LSTM designed to capture long-term dependencies and contextual information in the sequences.
  - Both Teacher models are trained independently (using Generic dataset) and then transfer knowledge (basing on KD approach) to help Student model achieving high performance in predicting protein succinylation sites.
- ***Student model:***
  - The Student model is designed to be a hybrid architecture that combines the strengths of the two Teacher models. Specifically, it integrates a single-layer CNN1D and a single-layer Bi-LSTM to mimic and learn from the architectures of both Teacher models.
  - The CNN1D layer enables the Student model to extract spatial features similar to Teacher 1, while the Bi-LSTM layer captures sequence dependencies and contextual information as Teacher 2 does.
  - This hybrid design allows the Student model to effectively distill knowledge from both Teachers and generalize well with fewer computational resources.
- ***KD:***
  - After training, both Teacher models are used to extract essential knowledge (including feature representations and inter-feature relationships) and transfer it to the Student model through a KD mechanism.
  - This knowledge encompasses the key features learned by the Teachers and the relational knowledge between these features.
- ***Distillation Loss:***
  - In the KD model, the distillation loss was designed to transfer knowledge from two Teacher models to a single Student model. This loss quantifies the divergence between the output distributions of the Teachers and the Student, using Kullback–Leibler Divergence [26, 37].
  - The distillation losses are computed by comparing the softened probability distributions of the Teacher models (T_1_ and T_2_) and the Student model (S) using a temperature parameter τ. These distributions were computed via the *softmax* function applied to the logits divided by τ. The formulae for the two distillation losses were presented as Equations (1) and (2), as follows:
    (5) $$L_{\mathrm{distill}1}=L_{\mathrm{distill}}(\mathrm{softmax}(\frac{T_{1}}{\tau }),\mathrm{sofmax}(\frac{S}{\tau })).\tau^{2}$$
     
    (6) $$L_{\mathrm{distill}2}=L_{\mathrm{distill}}(\mathrm{softmax}(\frac{T_{2}}{\tau }),\mathrm{sofmax}(\frac{S}{\tau })).\tau^{2}$$
  - The total loss of predictive models was designed to combine the classification loss of the Student model and the distillation losses from both Teacher models (Equation (3)). This combination ensures that the Student model learns effectively from the ground truth labels as well as the knowledge provided by the Teacher models.
    (7) $$L_{\mathrm{total}}=\alpha .L_{\mathrm{student}}+(1-\alpha )\cdot (L_{\mathrm{distill}1}+L_{\mathrm{distil}2})$$
    Wherein:
  - T_1_ and T_2_ are the logits output by Teacher 1 and Teacher 2, respectively.
  - S is the logits output by the Student model.
  - τ is the temperature parameter that controls the softness of the probability distributions. A higher τ produces a softer probability distribution, which facilitates the transfer of knowledge from Teachers to the Student.
  - L_distill_ refers to a distance measure, typically Kullback–Leibler Divergence, used to compute the discrepancy between the softened distributions.
  - The multiplication by τ^2^ ensures that the gradient magnitudes remain consistent as the temperature changes, as the gradients scale with τ.
  - L_student_: The classification loss of the Student model on the original labeled data (typically cross-entropy loss).
  - α: A weighting factor that balances the contribution of the classification loss and the distillation losses.

## Results and discussion

### Impact of word frequency in the training dataset on protein language models

To assess the impact of word frequency in the training dataset on species-specific datasets, we used TwoSampleLogo [38] to plot the cumulative percentage of residues. In this context, the term ‘word frequency’ refers to the frequency of amino acid occurrence (i.e., token frequency) in the protein sequences within the training dataset. Specifically, we calculated how often each amino acid appears in the training dataset, and we used this data to visualize its distribution and impact on model performance.

Based on the TwoSampleLogo plots, the cumulative percentages of over- and under-represented residues are displayed along the Y-axis. The letters above and below the X-axis represent frequently observed residues.

**Figure 2.**
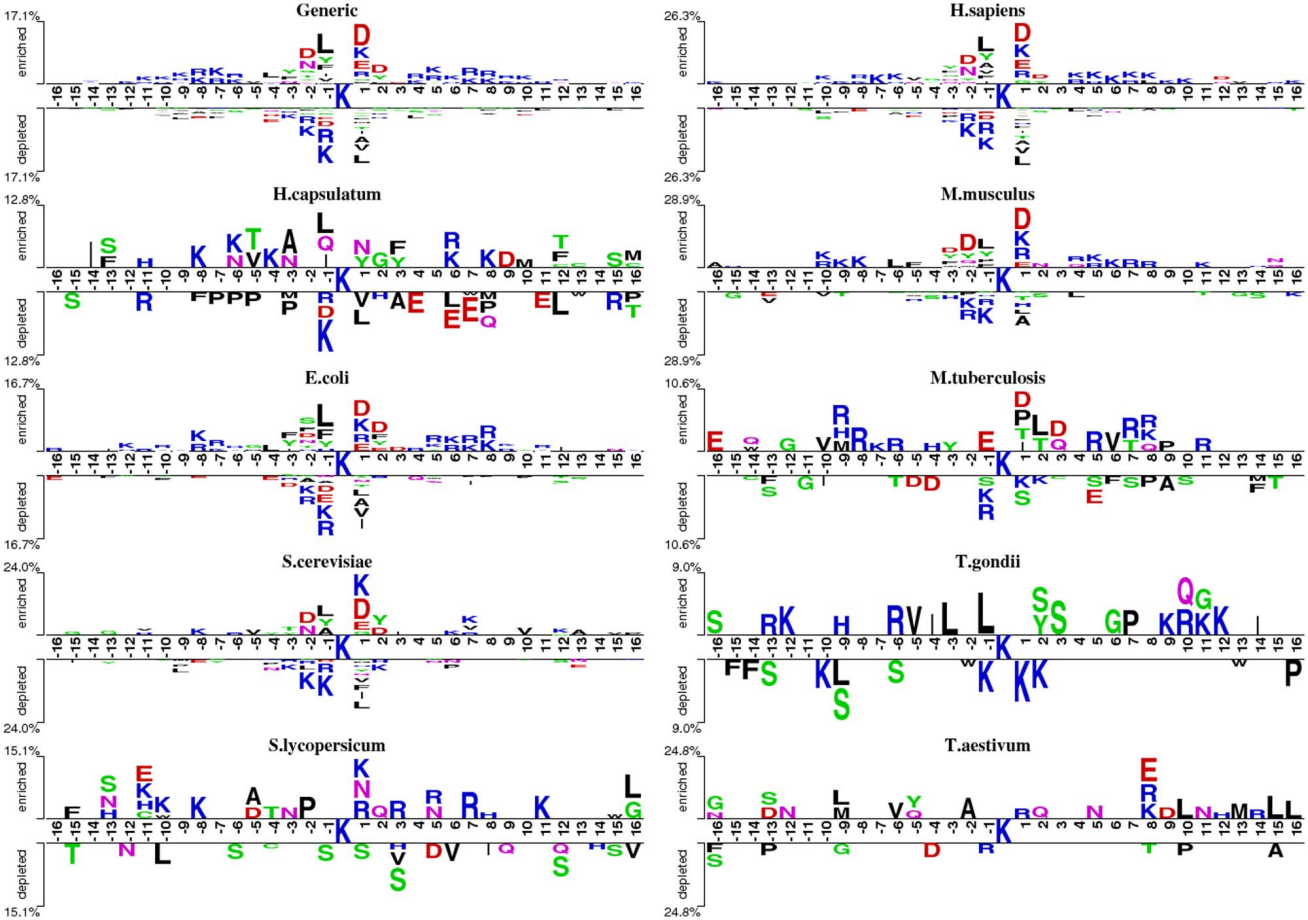
Two sample logo of generic and multiple-species

As shown in Fig. 2, the sequence patterns of *Generic, H. sapiens, S. cerevisiae, E. coli, and M. musculus* are highly similar, indicating a high probability that the model trained on the Generic dataset can be effectively generalized to predict these species. Fortunately, as displayed in Tables 4 and 5, the proposed model (**KD_MultiSucc**) trained on the Generic dataset provided a significant improvement in predictive performance, achieving higher MCC values on different species-specific datasets.

Our results demonstrate that the Generic prediction model can be effectively generalized to other species, achieving consistent and reliable results across diverse datasets while overcoming the limitations of species-specific training. These findings highlight the robustness and generalization capability of the proposed model when trained on a diverse and comprehensive dataset such as Generic.

### Effect of α and τ in KD

To investigate the impact of the distillation hyperparameters α (balancing factor between soft and hard losses) and τ (temperature for softening the teacher's output), we conducted an ablation study using the KD3 configuration as a representative case. The goal was to determine which values of α and τ would yield optimal performance.

Referring to Hinton *et al*. [26] and considering the specific characteristics of our proposed model, we varied *α* within the set [0.3, 0.5, 0.7] and *τ* within the set [3, 5, 10], and investigated the Student model’s performance on the Generic set. As shown in Table 6, the best-performing combination of *α* and *τ* was 0.5 and 10, respectively, which enabled the proposed model to achieve the highest performance in both cross-validation and independent testing. This finding validates our choice and demonstrates the effectiveness of our proposed model and the selected distillation parameters.

**Table 6.** Effect of α and τ on knowledge distillation.

Values of α and τ (in Knowledge Distillation)		Cross-validation		Independent testing	
α	τ	ACC	MCC	ACC	MCC
0.3	3	0.72	0.42	0.79	0.27
0.5	3	0.73	0.44	0.78	0.26
0.7	3	0.71	0.42	0.78	0.26
0.3	5	0.76	0.46	0.80	0.28
0.5	5	0.74	0.45	0.80	0.29
0.7	5	0.75	0.45	0.80	0.27
0.3	10	0.76	0.45	0.81	0.29
**0.5**	**10**	**0.77**	**0.47**	**0.81**	**0.32**
0.7	10	0.76	0.45	0.80	0.28

Bold values indicate the highest ones.

### Performance evaluation by cross-validation approach

As mentioned above, to determine the best architecture for predicting succinylation across various species, we constructed and evaluated four different architectures in this study *(Detailed information and its parameters are summarized in Table 4).* As displayed in Tables 7 and 8, the 10-fold cross-validation results indicate that the KD3 model consistently outperforms the others on both large-scale and small training datasets, achieving the highest values for ACC and MCC. On large-scale and relatively balanced datasets such as Generic, *E. coli*, and *S. cerevisiae*, KD3 achieves a strong trade-off between specificity and sensitivity (e.g., Generic: SPE = 0.84 ± 0.013, SEN = 0.62 ± 0.031) with low standard deviation (SD), reflecting stable learning and effective class separation.

**Table 7.** Performance evaluated by 10-fold cross-validation of the predictive models on all training datasets (metrics: SPE, SEN).

Dataset	KD1 ± SD		KD2 ± SD		KD3 ± SD		KD4 ± SD	
	SPE	SEN	SPE	SEN	SPE	SEN	SPE	SEN
Generic	0.85 ± 0.012	0.59 ± 0.041	0.84 ± 0.015	0.60 ± 0.035	**0.84 ± 0.013**	**0.62 ± 0.031**	0.81 ± 0.023	0.61 ± 0.041
*H. sapiens*	0.85 ± 0.025	0.57 ± 0.067	0.83 ± 0.027	0.61 ± 0.062	**0.86 ± 0.025**	**0.62 ± 0.058**	0.80 ± 0.031	0.60 ± 0.064
*H. capsulatum*	0.84 ± 0.045	0.40 ± 0.115	0.79 ± 0.062	0.42 0.153	**0.86 ± 0.042**	**0.50 ± 0.100**	0.82 ± 0.052	0.43 ± 0.127
*M. musculusis*	0.88 ± 0.047	0.43 ± 0.125	0.82 ± 0.056	0.52 ± 0.121	**0.84 ± 0.037**	**0.58 ± 0.101**	0.79 ± 0.089	0.56 ± 0.145
*E. coli*	0.87 ± 0.021	0.59 ± 0.063	0.84 ± 0.035	0.65 ± 0.048	**0.86 ± 0.022**	**0.63 ± 0.048**	0.78 ± 0.036	0.62 ± 0.052
*M. tuberculosis*	0.87 ± 0.048	0.25 ± 0.068	0.81 ± 0.069	0.37 ± 0.065	**0.82 ± 0.046**	**0.38 ± 0.055**	0.79 ± 0.066	0.40 ± 0.060
*S. cerevisiae*	0.85 ± 0.033	0.54 ± 0.065	0.82 ± 0.045	0.64 ± 0.067	**0.86 ± 0.023**	**0.60 ± 0.056**	0.80 ± 0.024	0.68 ± 0.075
*T. gondii*	0.71 ± 0.088	0.50 ± 0.123	0.75 ± 0.071	0.41 ± 0.124	**0.81 ± 0.061**	**0.42 ± 0.121**	0.73 ± 0.096	0.43 ± 0.134
*S. lycopersicum*	0.78 ± 0.085	0.42 ± 0.110	0.75 ± 0.067	0.47 ± 0.102	**0.87 ± 0.057**	**0.55 ± 0.098**	0.78 ± 0.082	0.46 ± 0.109
*T. eastivum*	0.89 ± 0.083	0.29 ± 0.159	0.76 ± 0.085	0.33 ± 0.157	**0.86 ± 0.077**	**0.44 ± 0.135**	0.70 ± 0.095	0.45 ± 0.144

Bold values indicate the highest ones.

**Table 8.** Performance evaluated by 10-fold cross-validation of the predictive models on all training datasets (metrics: ACC, MCC).

Dataset	KD1 ± SD		KD2 ± SD		KD3 ± SD		KD4 ± SD	
	ACC	MCC	ACC	MCC	ACC	MCC	ACC	MCC
**Generic**	0.76 ± 0.011	0.45 ± 0.025	0.76 ± 0.010	0.45 ± 0.025	**0.77 ± 0.009**	**0.47 ± 0.024**	0.72 ± 0. 012	0.42 ± 0.029
*H. Sapiens*	0.76 ± 0.0019	0.44 ± 0.051	0.77 ± 0.018	0.46 ± 0.048	**0.78 ± 0.017**	**0.49 ± 0.048**	0.71 ± 0.028	0.40 ± 0.049
*H. capsulatum*	0.69 ± 0.053	0.27 ± 0.109	0.67 ± 0.058	0.22 ± 0.125	**0.74 ± 0.050**	**0.38 ± 0.106**	0.69 ± 0.072	0.26 ±0.081
*M. musculusis*	0.73 ± 0.059	0.35 ± 0.134	0.72 ± 0.050	0.35 ± 0.133	**0.75 ± 0.040**	**0.43 ± 0.127**	0.74 ± 0.051	0.37 ± 0.131
*E. coli*	0.78 ± 0.022	0.48 ± 0.045	0.77 ± 0.024	0.49 ± 0.047	**0.79 ± 0.020**	**0.51 ± 0.042**	0.70 ± 0.057	0.38 ± 0.052
*M. tuberculosis*	0.66 ± 0.025	0.15 ± 0.041	0.66 ± 0.021	0.19 ± 0.037	**0.67 ± 0.020**	**0.22 ± 0.034**	0.64 ± 0.027	0.18 ± 0.038
*S. cerevisiae*	0.74 ± 0.032	0.41 ± 0.032	0.76 ± 0.028	0.46 ± 0.033	**0.77 ± 0.027**	**0.48 ± 0.032**	0.76 ± 0.028	0.46 ± 0.033
*T. gondii*	0.64 ± 0.048	0.21 ± 0.108	0.64 ± 0.048	0.16 ± 0.102	**0.68 ± 0.047**	**0.25 ± 0.098**	0.63 ± 0.057	0.15 ± 0.112
*S. lycopersicum*	0.66 ± 0.072	0.20 ± 0.135	0.66 ± 0.073	0.23 ± 0.125	**0.76 ± 0.053**	**0.44 ± 0.127**	0.67 ± 0.070	0.24 ± 0.122
*T. eastivum*	0.69 ± 0.078	0.23 ± 0.125	0.61 ± 0.082	0.09 ± 0.131	**0.72 ± 0.072**	**0.33 ± 0.124**	0.63 ± 0.085	0.23 ± 0.125

Bold values indicate the highest ones.

In contrast, on very limited training datasets, such as *M. musculus* (414 positive samples), *H. capsulatum* (332 positive samples), *T. gondii* (282 positive samples), and *T. aestivum* (113 positive samples), most models exhibit a drop in sensitivity due to the scarcity of positive sites. Nonetheless, KD3 maintains higher sensitivity compared to the other models (e.g., *M. musculus*: SEN = 0.58 ± 0.101 for KD3 versus 0.43 ± 0.125 for KD1), indicating a better ability to learn from limited data.

Furthermore, KD3 generally shows lower or comparable SD values across datasets, suggesting greater consistency and generalization ability. Overall, these results demonstrate that KD3 is more robust and better suited for handling data imbalance and varying dataset sizes.

The KD3 model outperforms KD1 and KD2 in cross-validation tests, as indicated by the ACC and MCC metrics. This superior performance is due to KD3’s hybrid architecture that combines CNN and Bi-LSTM, whereas KD1 and KD2 utilize only one of the two networks (either CNN1D or Bi-LSTM). As a result, KD1 and KD2 do not leverage the ability to combine information from both teachers, which limits their learning performance.

Although KD3 and KD4 share similar architectures that integrate CNN1D and Bi-LSTM in the Student model, the main difference lies in the order of learning. KD3 applies CNN1D first, followed by Bi-LSTM (CNN1D→Bi-LSTM), facilitating the extraction of local features from the protein sequence before modeling long-range dependencies. This approach enables the model to better capture local motifs and enhances its ability to integrate global information from Bi-LSTM.

**Figure 3.**
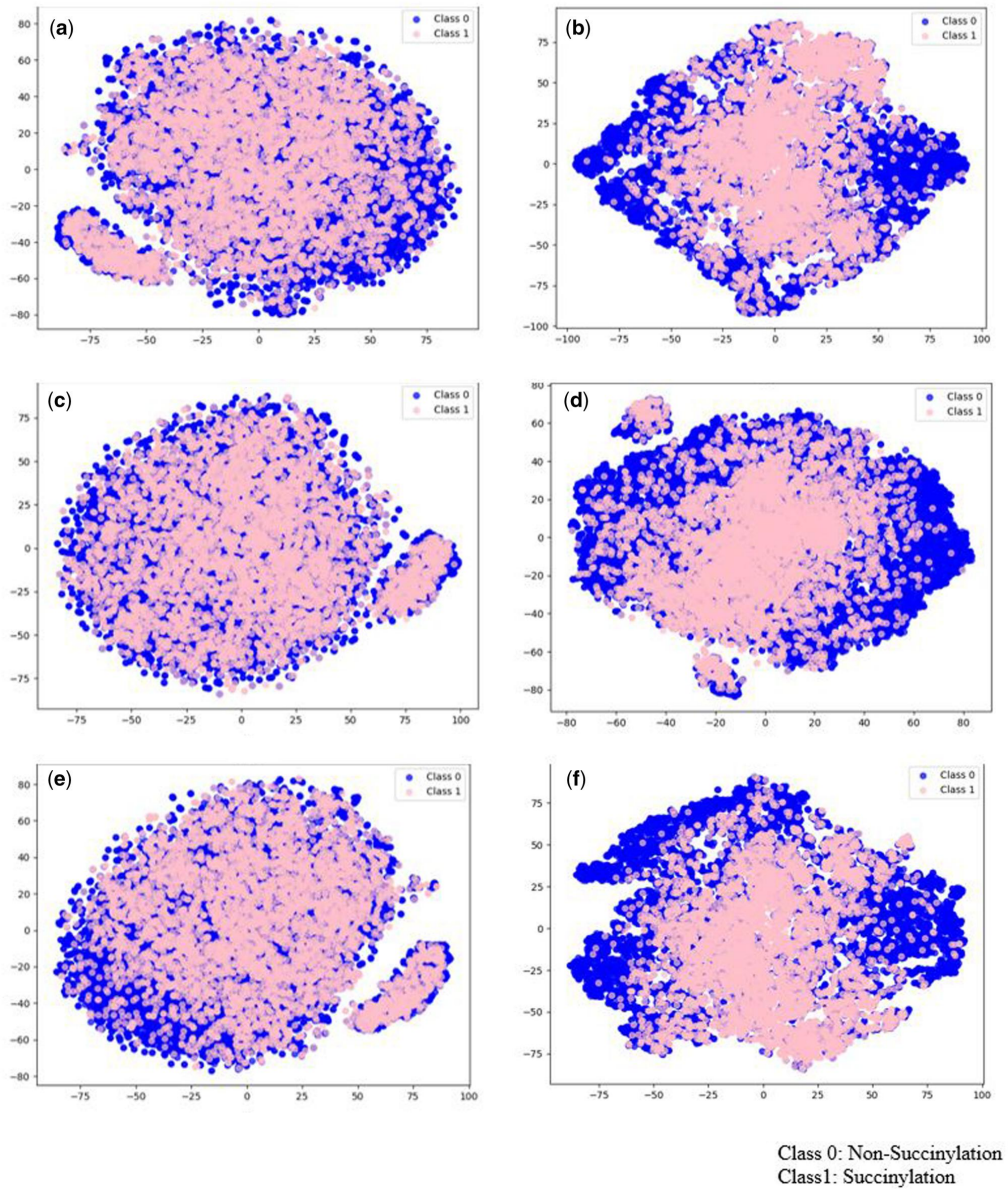
t-SNE visualization of the model before training by different of models on Generic dataset: (a) before training Teacher 1 model, (b) after training teahcer 1 model, (c) before training Teacher 2 model, (d) after training Teahcer 2 model, (e) before training Student model, (f) after training Student model using Knowlege distillation

**Figure 4.**
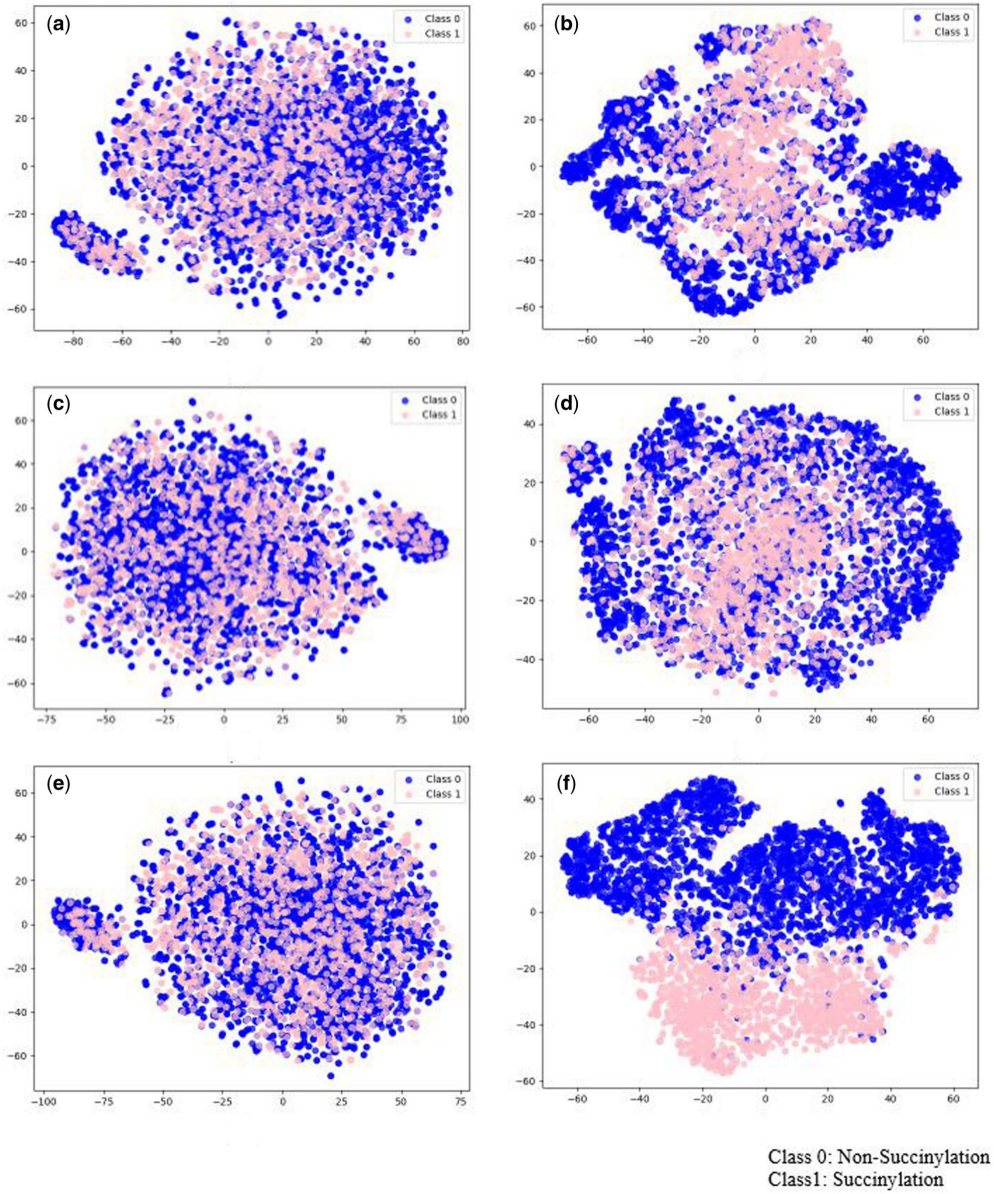
t-SNE visualization of the model before training by different of models on *H.sapiens* dataset. (a) before training Teacher 1 model, (b) after training Teahcer 1 model, (c) before training Teacher 2 model, (d) after training teahcer 2 model, (e) before training Student model, (f) after training Student model using Knowlege distillation

In contrast, KD4 reverses this order (Bi-LSTM→CNN1D), with Bi-LSTM learning long-range dependencies before processing local features. This can lead to the loss of important local features, resulting in lower performance compared to KD3. This difference in learning order explains why KD3 outperforms KD4 and the other models in predicting succinylation. Fortunately, as shown in Tables 3, 7, and 8, the KD3 achieves highest performance, reaching the highest values of MCC on all dataset. As a result, KD3 was selected as the final model for predicting protein succinylation in this study. To further assess the quality of the learned feature representations, we applied t-SNE [29] to project the high-dimensional feature vectors into two-dimensional space. As shown in Fig. 3 (Generic dataset) and Fig. 4 (*H. sapiens*), subfigures (a), (c), and (e) correspond to the feature distributions before training for Teacher 1, Teacher 2, and the Student model, respectively. In these cases, the feature embeddings of succinylated and non-succinylated sites are highly overlapping, indicating that the models had not yet learned class-discriminative features.

In contrast, subfigures (b), (d), and (f) illustrate the distributions after training. Here, the feature representations become noticeably more separated between the two classes, particularly in the Student model, suggesting that both Teacher and Student models successfully captured informative patterns for succinylation site prediction. Moreover, the improvement observed in the Student model confirms that knowledge distilled from the Teacher models contributes to learning a more effective and separable latent space.

### Performance evaluation by independent testing

To examine the ability of the proposed model, an independent testing approach was performed in this study. The independent testing was conducted on all individual species-specific datasets. As shown in Table 9, KD3 outperforms KD1, KD2, and KD4 on all species-specific datasets, achieving the highest MCC values. This indicates that KD3 is appeared to be the optimal KD architecture for this study.

**Table 9.** The result of independent test of the predictive models on all independent testing datasets.

Dataset	KD1				KD2				KD3				KD4			
	SPE	SEN	ACC	MCC	SPE	SEN	ACC	MCC	SPE	SEN	ACC	MCC	SPE	SEN	ACC	MCC
**Generic**	0.84	0.58	0.82	0.29	0.83	0.63	0.81	0.30	**0.83**	**0.66**	**0.81**	**0.32**	0.83	0.65	0.81	0.31
***H. Sapiens***	0.82	0.76	0.82	0.23	0.80	0.81	0.80	0.24	**0.85**	**0.76**	**0.85**	**0.26**	0.84	0.76	0.84	0.25
*H. capsulatum*	0.83	0.28	0.79	0.08	0.71	0.50	0.69	0.12	**0.71**	**0.60**	**0.71**	**0.18**	0.76	0.46	0.74	0.14
*M. musculusis*	0.81	0.58	0.80	0.17	0.78	0.54	0.77	0.14	**0.79**	**0.63**	**0.78**	**0.18**	0.83	0.54	0.82	0.17
***E. coli***	0.85	0.57	0.80	0.39	0.81	0.60	0.77	0.35	**0.84**	**0.64**	**0.80**	**0.41**	0.83	0.57	0.79	0.36
*M. tuberculosis*	0.86	0.23	0.74	0.10	0.86	0.28	0.74	0.15	**0.79**	**0.39**	**0.71**	**0.17**	0.78	0.39	0.70	0.16
***S. cerevisiae***	0.85	0.58	0.83	0.27	0.77	0.64	0.77	0.23	**0.84**	**0.67**	**0.83**	**0.31**	0.78	0.66	0.77	0.24
*T. gondii*	0.68	0.42	0.66	0.06	0.80	0.38	0.76	0.13	**0.79**	**0.42**	**0.76**	**0.15**	0.81	0.39	0.77	0.14
*S. lycopersicum*	0.81	0.27	0.76	0.07	0.62	0.48	0.60	0.06	**0.62**	**0.61**	**0.62**	**0.14**	0.68	0.42	0.66	0.07
*T. eastivum*	0.75	0.39	0.72	0.09	0.66	0.45	0.64	0.07	**0.71**	**0.52**	**0.69**	**0.14**	0.70	0.42	0.68	0.08

Bold values indicate the highest ones.

Furthermore, in order to evaluate the generalization ability of the optimal architecture (KD3), we decided to use the Generic dataset for model learning and to utilize it for cross-species prediction. As displayed in Table 10 the **KD_MultiSucc** model (i.e., the KD3 model trained using the Generic dataset) achieves a significant improvement of the predictive performance with higher values of MCC on all specific-species datasets. This result underscores the model’s reliability and generalization for cross-species prediction of protein succinylation sites. Consequently, the **KD_MultiSucc** is selected as final model for cross-species prediction of protein succinylation sites.

**Table 10.** The performance of KD_MultiSucc (i.e., the KD3 model trained using the Generic dataset) in predicting species-specific datasets.

Specific-species datasets	SPE	SEN	ACC	MCC	MCC improvement (compared to model training on specific-species dataset)
*H. Sapiens*	0.93	0.91	0.92	0.45	+0.19
*H. capsulatum*	0.72	0.72	0.72	0.25	+0.07
*M. musculus*	0.87	0.83	0.87	0.36	+0.18
*E. coli*	0.84	0.84	0.84	0.58	+0.17
*M. tuberculosis*	0.86	0.33	0.75	0.20	+0.03
*S. cerevisiae*	0.85	0.94	0.86	0.48	+0.17
*T. gondii*	0.79	0.69	0.78	0.31	+0.16
*S. lycopersicum*	0.61	0.76	0.63	0.23	+0.09
*T. eastivum*	0.68	0.63	0.68	0.18	+0.04

### Comparison with other similar predictors

To examine the robustness and practical ability of our proposed model, we compared its performance with that of existing predictors specifically designed for predicting succinylation sites. In this study, the most widely and up-to-date predictors, GPSuc and SuccinSite 2.0, DeepSuccinylSite, and LMSuccSite have been selected for the comparison. As shown in Table 11, the proposed model, KD_MultiSucc, outperforms GPSuc, SuccinSite 2.0, and DeepSuccinylSite with the highest MCC values on both specific-species datasets. Although our model’s performance is slightly lower (0.04 smaller in MCC value) than that of LMSuccSite, it has a much smaller size (in terms of parameters and memory, as shown in Table 12) than LMSuccSite. Besides, the KD_MultiSucc provides a novel architecture designed for effectively prediction of succinylation sites. This result demonstrates superior performance, particularly for challenging species, making it a robust tool for succinylation site prediction.

**Table 11.** Performance comparison of the proposed model **KD_MultiSucc** (trained on Generic dataset) with other similar predictors across multiple specific-species dataset.

Dataset	KD_MultiSucc		GPSuc		SuccinSite 2.0		DeepSuccinylSite [11]		LMSuccSite [20]	
	ACC	MCC	ACC	MCC	ACC	MCC	ACC	MCC	ACC	MCC
Generic	0.81	0.32	0.88	0.28	0.87	0.24	0.70	0.27	0.79	**0.36**
*H. sapiens*	0.92	**0.45**	0.87	0.28	0.87	0.24	–	–	–	–
*H. capsulatum*	0.72	**0.25**	–	–	–	–	–	–	–	–
*M. musculusis*	0.87	**0.36**	0.78	0.15	0.77	0.10	–	–	–	–
*E. coli*	0.84	**0.58**	0.71	0.25	0.69	0.19	–	–	–	–
*M. tuberculosis*	0.75	**0.20**	–	–	0.66	0.14	–	–	–	–
*S. cerevisiae*	0.86	**0.48**	0.81	0.25	0.81	0.22	–	–	–	–
*T. gondii*	0.78	**0.31**	0.80	0.30	0.79	0.19	–	–	–	–
*S. lycopersicum*	0.63	**0.23**	0.80	0.22	0.77	0.17	–	–	–	–
*T. eastivum*	0.68	**0.18**	–	–	–	–	–	–	–	–

Bold values indicate the highest ones.

**Table 12.** Comparison of total parameters, memory inn Generic dataset

Tools	Features	Size of Features	Algorithm	Available of codes and resource	Total parameters	Sum of memory (KB)
KD_MultiSucc	Embedding layer	300	Knowledge distillation (CNN1D, Bi-LSTM)	Github	117,802	46,016
DeepSuccinylSite [11]	One-hot and Word2vec embedding	–	CNN2D	Parameter public in paper	1,850,446	722,947
LMSuccSite [20]	PRotT5 + Embedding layer	1024 + 1024	ANN+CNN2D	Github	186,450	73,223
GPSuc	AAIndex, BE, AAC, PSSM, and pCKSAAP	–	RF	Sever	–	–
SuccinSite 2.0	pCKSAAP, Binary		RF	Sever	–	–

## Conclusion

In this study, we present a predictor called **KD_MultiSucc** for cross-species prediction of succinylation sites using Multi-Teacher KD and Word Embedding. The proposed predictor was trained using the Generic dataset and demonstrated its effectiveness in predicting succinylation sites across multiple species. By leveraging the strengths of KD, **KD_MultiSucc** successfully generalized predictions, particularly for species with similar sequence patterns, such as *H. sapiens*, *S. cerevisiae*, *E. coli*, and *M. musculus*. Cross-validation evaluation revealed that the proposed predictor is a valuable approach for predicting protein succinylation sites with high performance. Furthermore, independent testing, including comparisons with similar existing predictors, demonstrated the model’s generalization ability and superior performance in predicting protein succinylation sites.

## Data Availability

Data are available at https://github.com/nuinvtnu/KD_MultiSucc/.
